# Multifunctional Magnetic Droplet Robots for Urological Applications: From Drug Delivery to Stone Retrieval

**DOI:** 10.3390/mi17050569

**Published:** 2026-05-03

**Authors:** Angelina Lin, Joanna Tang, Chunlian Zhong, Shanshan Yao, Zhaoqing Cong

**Affiliations:** 1 Department of Urology, Stony Brook University, Stony Brook, NY 11794, USA; angelina.lin@stonybrook.edu (A.L.); joanna.tang@stonybrook.edu (J.T.); chunlian.zhong@stonybrookmedicine.edu (C.Z.); 2 Stony Brook Cancer Center, Renaissance School of Medicine, Stony Brook University, Stony Brook, NY 11794, USA; 3 Department of Mechanical Engineering, Stony Brook University, Stony Brook, NY 11794, USA; shanshan.yao@stonybrook.edu

**Keywords:** droplet robots, programmable electromagnetic fields, 3D-printed urinary tract models, drug delivery, stone retrieval

## Abstract

Therapeutic interventions within the urinary system are often limited by the complex and tortuous anatomy of the renal pelvis and ureters, restricting access to deep regions and increasing the risk of mucosal trauma. In this study, we present a multifunctional, magnetically controlled ferrofluid droplet robotic platform engineered for high deformability and precision navigation. A custom electromagnetic actuation system was developed and optimized via COMSOL Multiphysics (version 6.3, COMSOL Inc., Stockholm, Sweden) simulations to generate programmable magnetic fields. Experimental validation in both simplified environments and anatomically realistic 3D-printed urinary tract models demonstrated the droplets’ capacity for controlled locomotion, reversible deformation, and traversing constrictions significantly smaller than their resting diameter. The droplets’ locomotion and extreme deformability are governed by the dynamic balance between the applied magnetic gradient forces, the restoring interfacial tension of the ferrofluid, and the fluidic viscous drag. Quantitatively, the droplets achieved robust translational velocities up to 260 mm/s under single-coil actuation (51 mT, 20 Hz) and 108 mm/s under a more stable dual-coil configuration (51 mT, 8.3 Hz). Furthermore, two clinically relevant functionalities were successfully executed: rapid vibration-induced release of encapsulated dye for targeted drug delivery, and the precise mechanical capture and transport of artificial kidney stones. These results establish a highly versatile platform for minimally invasive urological procedures, highlighting the immense potential of soft magnetic microrobotics for integrated therapeutic applications.

## 1. Introduction

The urinary system, including the kidneys, ureters, bladder, and urethra, functions not only as the primary drainage pathway for metabolic waste elimination, but also as a critical regulator of homeostasis. It maintains blood volume and pressure, modulates electrolyte balance, and stabilizes blood pH [1,2,3]. Urological diseases, such as urinary tract infection, urinary cancer, urolithiasis, and chronic kidney disease, affect over 20% of the population in the United States [4,5,6]. Their high prevalence and cost of prevention make them major global public health issues. However, the normal voiding-mediated washout effect and the inherent physiological barrier of the urinary tract, characterized by a physical shield formed by a tight cell layer structure and chemical defense mediated by the glycosaminoglycan composition [7,8], significantly hinders drug retention and penetration, reducing therapeutic efficacy [9]. The conventional drug delivery method—intravesical instillation—is limited by rapid clearance during voiding and by physiological drug absorption into the urothelium [10,11,12]. Additionally, narrow, curved, and highly constrained anatomical characteristics dramatically restrict localized therapeutic interventions [13]. Conventional tools such as ureteroscopes and endoscopic instruments are constrained by their rigid or semi-flexible geometries, which can hinder access to deep renal structures and increase the risk of mucosal trauma [14].

The development of magnetic microrobotics offers new possibilities for targeted drug delivery and therapy [15,16,17,18]. These microrobots can actively penetrate deep tissue and be accurately located under electromagnetic control. Therefore, they can effectively overcome the washout effect and physiological barriers of the urinary tract and precisely deliver drugs to a narrow lesion without damaging normal tissues [19,20,21]. Compared with conventional drug delivery methods and interventions in the urinary system, unique advantages, including wireless control, fuel-free locomotion, precise manipulation, and deep tissue penetration [22,23], make electromagnetic-actuated systems well-fit for urological disease treatment. For example, Fe_3_O_4_-based magnetic microrobots can effectively locomote through the complex urinary anatomy and achieve targeted drug delivery [15,16]. Following that, magnetic microrobots have been designed as retrievable platforms to minimize long-term systemic toxicity, such as recovered devices [24] and microrobots [25,26] that were developed to precisely deliver drugs to lesions and subsequently be retrieved using controlled electromagnetic fields. The integration of droplet technologies further evolves magnetic microrobotics applications. The programmable electromagnetic control can achieve droplet multi-manipulations, including continuous liquid-phase deformability, reconfigurability, and the ability to dynamically split and merge, allowing locomotion in the narrow, curved, highly constrained biological environment. As a consequence of these advantages, they can navigate through the narrow and curved urinary tract and release drugs to the targeted lesions [27].

Despite these conceptual advantages, translating droplet microrobotics into practical urological interventions remains challenging. Most existing magnetic droplet platforms are evaluated in simplified 2D planar environments or simple straight tubes, which fail to replicate the complex, non-linear 3D topology of the human urinary tract [28,29,30]. Furthermore, achieving a highly integrated platform capable of both on-demand chemical therapeutic release (drug delivery) and forceful mechanical tasks (stone retrieval) using a single untethered soft robot has rarely been demonstrated.

Herein, a multifunctional, programmed ferrofluid droplet robotic platform is designed for minimally invasive urological applications. By integrating a custom-built electromagnetic actuation array guided by Multiphysics simulations, the spatial morphology, locomotion speed, and navigation trajectory of these droplet robots can be precisely modulated. Importantly, we validate the precise spatiotemporal navigation and extreme deformability of the droplets not only in highly constricted variable-width channels but also within an anatomically realistic 3D-printed human urinary tract model. Beyond agile locomotion, we demonstrate the platform’s versatility through vibration-triggered localized drug release and magnetic “capture-and-carry” execution for artificial kidney stone retrieval. This work establishes a viable engineering framework for next-generation soft magnetic robots in complex clinical environments.

## 2. Materials and Methods

### 2.1. Electromagnetic Actuation System

**Figure 1:**
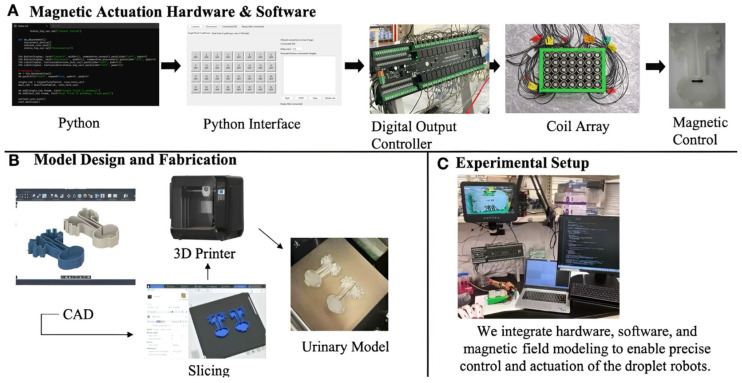
The design of the electromagnetic actuation system and function evaluation. (**A**) System control workforce: Python (version 3.12) interface to digital output controller to coil array, enabling magnetic manipulation of the droplet robot. (**B**) The fabrication of the mimicry urinary system: CAD modeling in Autodesk Fusion 360, slicing, and 3D printing of the anatomically inspired urinary model. (**C**) The scheme of the overall experimental setup illustrating the integration of hardware and the fluid medium.

The experimental platform was designed focusing on a multi-stage control and fabrication workflow. This electromagnetic actuation system (Figure 1A) utilized a custom Python-to-Python (Python 3.12) interface to obtain precise navigation. These signals were subsequently transmitted to a digital output controller, which powered the physical coil array. This coordinated electrical system allowed for the precise magnetic control of the ferrofluid droplet robot as it locomoted through the 3D urinary model. The full implementation of the control software and graphical user interface is available in a public repository (please refer to https://github.com/joannatang-a11y/coil-array-controller (accessed on 1 April 2026).

The fabrication of the testing environment follows a dedicated digital-to-physical pipeline (Figure 1B). The anatomical structures were designed using Autodesk Fusion 360 (version 2.0, Autodesk, San Rafael, CA, USA), ensuring precise geometric mimicry of the renal pelvis and ureters. Followed slicing with QIDI studio (version 2.5.2.50), these urinary models were printed by a QIDI Q1 pro 3D printer (QIDI Technology, Ruian, Zhejiang, China). This 3D urinary model provides a possible way to evaluate the function of the electromagnetic actuation system in an emulational urinary system.

The overall experimental setup (Figure 1C) integrates these electromagnetic control systems and fabrication components into a unified workspace, where the droplets’ movement within the 3D-printed urinary system is monitored under controlled conditions.

### 2.2. Numerical Modeling

Finite element analysis (FEA) was performed using COMSOL Multiphysics (version 6.3, COMSOL Inc., Stockholm, Sweden) to characterize the magnetic field distribution and spatial gradients generated by the electromagnetic coil array. Simulations were conducted for both single-coil and dual-coil actuation configurations. A grid independence study was performed comparing coarse, normal, fine, and finer mesh densities. The maximum magnetic flux density remained stable at 0.252 T across all meshes, and the final droplet displacement showed negligible variation (e.g., 0% change between normal and fine meshes in the single-coil model). Consequently, the fine mesh was utilized for all subsequent analyses to optimize the balance between computational efficiency and numerical accuracy (Tables S1 and S2).

### 2.3. Fabrication of Fluorescent-Dye-Loaded Ferrofluid Droplets

The magnetically controlled droplets were formulated using commercial EFH1 ferrofluid (FerroTec, Santa Clara, CA, USA), which consists of iron oxide nanoparticles at a nominal volume fraction of 7.9%. To ensure colloidal stability and prevent irreversible agglomeration during magnetic deformation, these nanoparticles were sterically stabilized with an oleic acid surfactant (15% *v*/*v*) and suspended in a light hydrocarbon/mineral oil carrier liquid (77.1% *v*/*v*).

The ferrofluid droplets were prepared and introduced into a controlled liquid environment consisting of 0.9% NaCl solution (pH = 6.0), selected to approximate the physiological ionic and slightly acidic pH conditions typically found in human urine. To reduce adhesion between the droplets and the model surface, the urinary tract model was coated with agarose hydrogel (1 g agarose in 100 mL deionized water), forming a hydrated, low-friction interface. For mimicking drug delivery experiments, a fluorescent dye solution was first prepared by dissolving a trace amount of Phloxine B (Spectrum Chemical, New Brunswick, NJ, USA) in an aqueous polyethylene glycol (PEG) 3350 (Ambeed, Arlington Heights, IL, USA) solution (3 g of PEG 3350 in 7 mL of deionized water). Subsequently, 50 μL of this dye solution was added into 500 μL of droplets and vortexed for 30 min to form fluorescent-dye-loaded ferrofluid droplets.

### 2.4. Drug Release Assay

To evaluate the drug release capability, 0.1 mL of fluorescent-dye-encapsulated ferrofluid droplets was added into the 3D urinary model containing 30 mL of NaCl solution under electromagnetic conditions. The locomotion and triggered release of fluorescent dye were precisely manipulated with programmed electromagnetic fields.

### 2.5. Stone Retrieval Assay

To verify the retrieval of stones from the urinary tract, we performed a precise electromagnetic control. These droplets were navigated to the targeted location under an electromagnetic field (51 mT, 8.3 Hz), and subsequently the stones were retrieved by a modulating electromagnetic field switch.

## 3. Results

### 3.1. Locomotion and Deformability Assays of Ferrofluid Droplets

**Figure 2:**
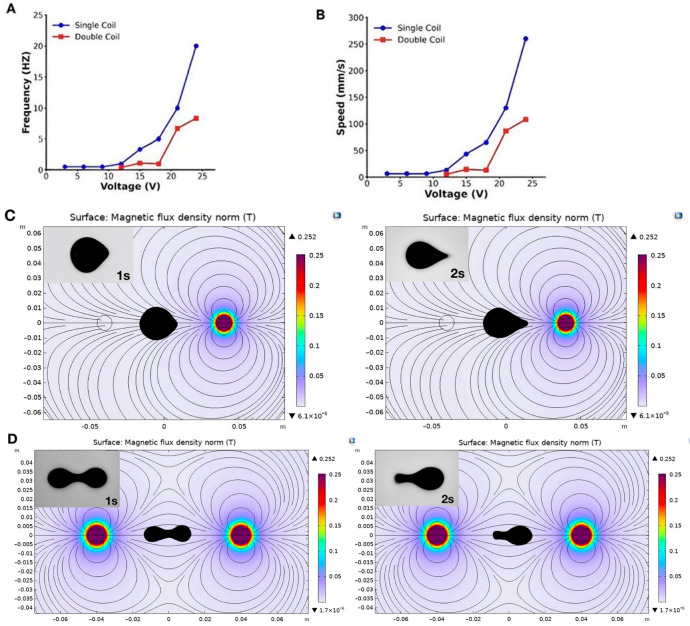
Droplet dynamic analyses under varying electrical inputs. (**A**) Single-coil actuation, showing a rapid increase in velocity with increasing voltage and achieving a maximum speed of 260 mm/s at 51 mT (20 Hz). (**B**) Dual-coil actuation, exhibiting a lower maximum velocity (108 mm/s at 51 mT, 8.3 Hz) but more stable and controlled motion across the tested voltage range. (**C**) A time-lapse sequence of single-coil locomotion, showing rapid propulsion and longitudinal elongation. (**D**) A time-lapse sequence of dual-coil locomotion, demonstrating more stable, controlled translational movement with a flattened morphology.

The actuation performance of the ferrofluid droplets was evaluated under two distinct modes: single-coil and dual-coil actuation (Movie S1). Quantitatively, droplet velocity showed a positive correlation with applied voltage (Figure 2A,B). Under single-coil actuation, the droplets achieved a maximum velocity of 260 mm/s (51 mT, 20 Hz). In contrast, dual-coil actuation resulted in a reduced maximum speed of 108 mm/s (51 mT, 8.3 Hz). To directly contextualize this kinematic performance, we compared our maximum translational velocity with representative magnetic droplet and microrobotic systems reported in the literature (Table 1). As shown, locomotion speeds across different platforms span several orders of magnitude, ranging from ~20 mm/s for conventional ferrofluid droplets to ~200–440 mm/s for highly agile liquid metal or advanced liquid microrobots. The velocities achieved by our ferrofluid droplet robot fall well within the upper echelon of current state-of-the-art systems. Notably, while many existing high-speed platforms rely on bulky, global multi-coil magnetic setups to achieve propulsion, our platform demonstrates comparable or superior agility utilizing a compact, programmable localized electromagnetic coil array.

Consistently, the behaviors of the droplets also varied significantly between these modes. As shown in Figure 2C, single-coil actuation produced a more concentrated magnetic field, causing the droplet to elongate significantly, achieving a maximum aspect ratio (length/width) of 1.54 as it was rapidly pulled toward the active center. This mode is optimal for high-speed transport through open channels. Conversely, dual-coil actuation (Figure 2D) generated a more distributed magnetic gradient, maintaining a more stable aspect ratio of approximately 2.76. This resulted in a flatter, more stable droplet profile with reduced peak acceleration but enhanced spatial control. This stability was critical for navigating the complex curvatures of the 3D-printed urinary model. Therefore, the dual-coil mode was selected for the following function evaluations. Despite these morphological changes, the droplets maintained structural integrity. This deformability capability allows them to locomote through a confined biological environment, which is even narrower than their static diameter without fragmentation.

### 3.2. Performance of Ferrofluid Droplets Through Electromagnetic Actuation

**Figure 3:**
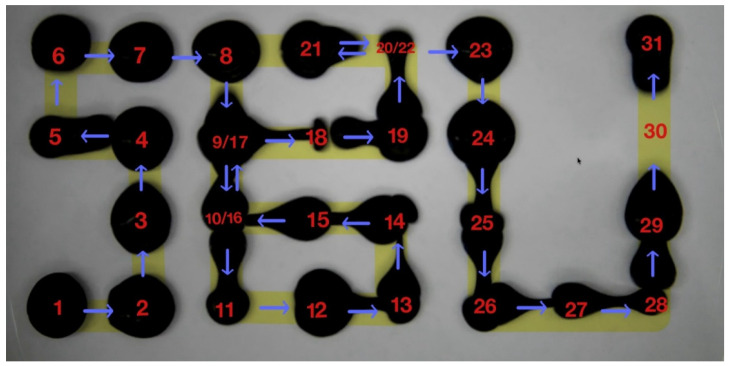
The programmable magnetic control of ferrofluid motion in the given trajectory, shown via time-lapse. The split and merge of the droplets along the given path eventually displayed the “SBU” acronym, illustrating that high-precision spatiotemporal control was enabled by the programmable coil array. The blue arrows indicate the direction of droplet movement, and the red numbers denote the chronological sequence of the droplets’ positions.

**Figure 4:**
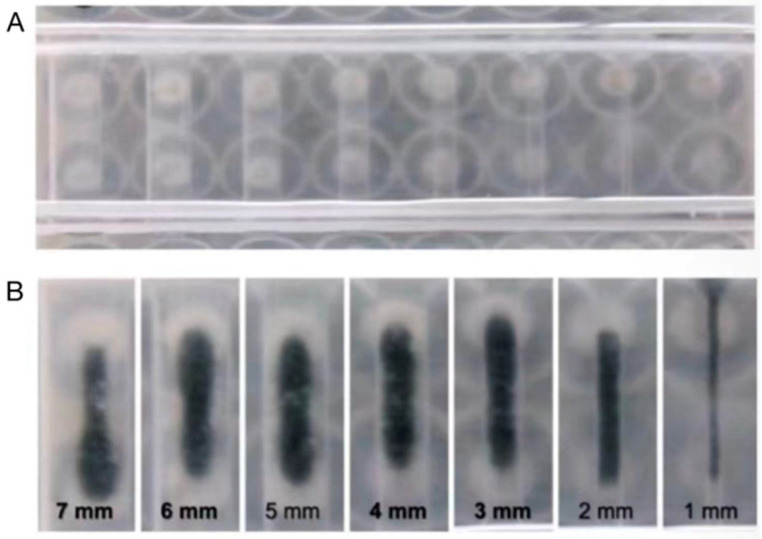
Deformation in confined geometries. (**A**) The experimental platform for variable-width testing. (**B**) Representative images showing the droplet elongating to traverse constrictions from 7 mm down to 1 mm.

For the evaluation of the electromagnetic actuation behaviors of the droplets, a 51 mT intensity and 8.3 Hz frequency of the magnetic field was used to manipulate the performance of the droplets. These droplets successfully locomoted following a complex, non-linear trajectory (1 → 2 → 3…… → 31). As shown in Figure 3 (Movie S2), the droplets were guided to trace the acronym “SBU,” symbolizing Stony Brook University. This demonstrated that the custom Python interface and electromagnetic coil array could execute intricate, multi-directional instructions with high fidelity. To consolidate the deformability of the droplets within a confined biological environment, the constricted locomotion was tested using a variable-width channel to simulate the physiological narrowing often encountered in the ureter. As shown in Figure 4 (Movie S3), the ferrofluid droplet was actuated through gradually narrower spaces, ranging from 7 mm down to 1 mm. At the narrowest 1 mm constriction, the droplet achieved an extreme aspect ratio of 38.5, representing an about nine-fold decrease compared to its static diameter. Despite the increased wall friction in the highly confined region, the droplet maintained continuous locomotion at a velocity of 73.64 mm/s without fragmentation. These results indicate a key advantage of liquid-phase robotics over rigid instruments: the droplet can achieve dynamical deformability, adopting a highly elongated morphology to locomote through narrow spaces significantly smaller than its static diameter. While the droplet underwent dramatic transformation as it navigated through the highly confined regions, it still maintained structural integrity without fragmentation. These targeted locomotion and adaptive deformability capabilities make it possible to integrate this system into future urological applications.

### 3.3. Precise Locomotion in a 3D Mimicking Urinary Model

**Figure 5:**
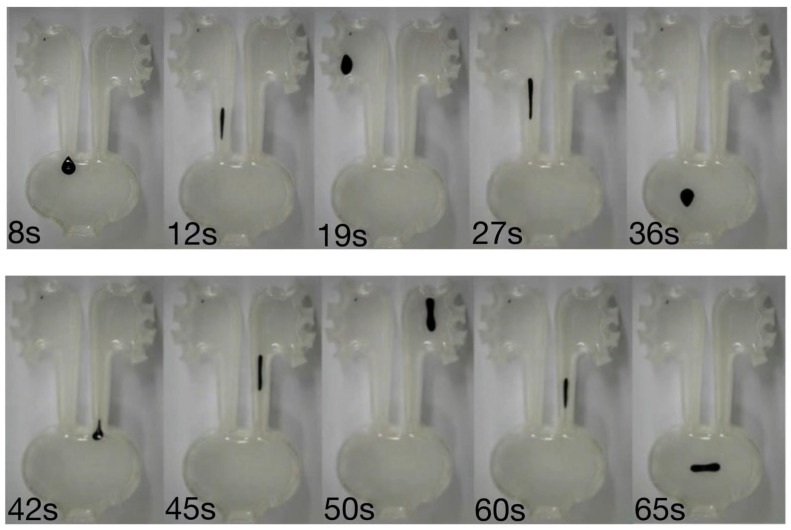
Magnetic droplet navigation within an anatomically inspired urinary tract model. A time-lapse sequence showing controlled droplet motion through curved and branching pathways under magnetic actuation, demonstrating the ability to traverse confined geometries mimicking the ureter and renal pelvis.

To evaluate the feasibility of these electromagnetically actuated droplets for complex interventions within the urinary system, droplet behaviors were assessed using a 3D mimicking urinary model. These droplets displayed a precise and stable locomotion capable of overcoming the complex 3D surface curvatures (Figure 5, Movie S4). Under the programmed actuation (51 mT, 8.3 Hz), the droplet successfully navigated from the simulated bladder region to the deep renal pelvis in around 11 s, maintaining an average navigation speed of 5.93 mm/s. Across four repeated trials, the droplet achieved a 100% success rate in reaching the targeted upper urinary tract branch. Consequently, the precise targeting and navigation along with the complex confined mimicking urinary model offers the potential for therapeutic applications in the narrow, curved, and highly constrained urinary system.

### 3.4. Targeted Drug Delivery and Stone Retrieval

**Figure 6:**
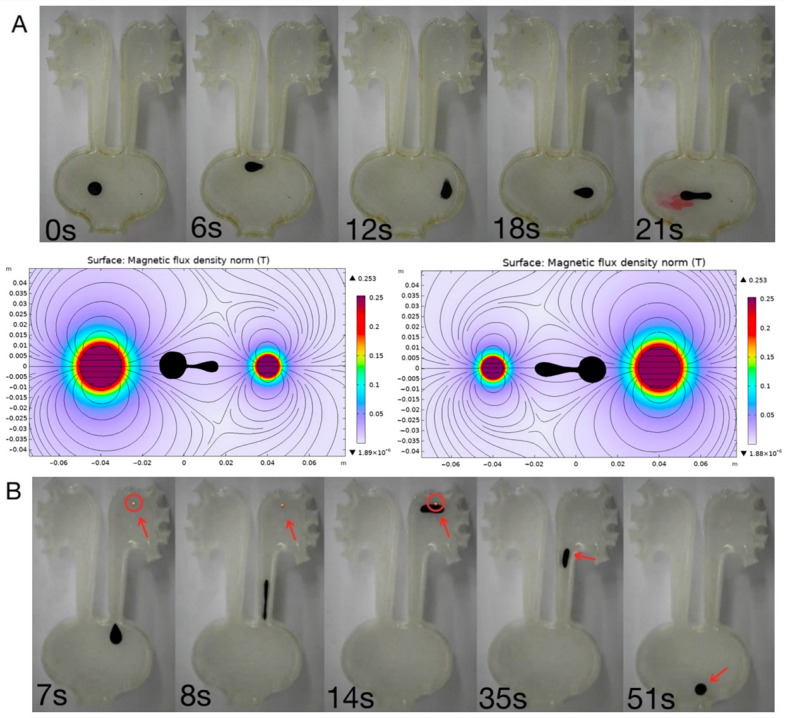
The multifunctional performance of the magnetic droplet robots: targeted drug delivery and stone retrieval. (**A**) The vibration-induced release of encapsulated dye, demonstrating localized payload delivery under magnetic actuation along with their COMSOL simulations. (**B**) Representative images showing the capture, encapsulation, and transport of artificial kidney stones, demonstrating the droplet’s mechanical “capture-and-carry” capability. The red arrows indicate the artificial stone locations.

Beyond locomotion and navigation, the ferrofluid droplets exhibited multifunctional capabilities, including targeted drug delivery and stone retrieval. Drug delivery was achieved through a vibration-assisted magnetic actuation mode (e.g., alternating frequency at 8.3 Hz at 51 mT), which effectively disrupted the dynamic interfacial tension of the droplet. Quantitative image intensity analysis revealed a rapid and highly localized release profile. Upon applying the vibratory magnetic field, most of the encapsulated fluorescent dye was explosively dispersed into the targeted region within just 0.25 s (Figure 6A, Movie S5). This rapid bolus release kinetics is highly advantageous for urological applications, as it can sustain a high localized drug concentration at the lesion site, significantly counteracting the dilution and rapid clearance caused by the continuous flow of urine. Additionally, the droplets displayed the retrievable ability to capture and transport artificial kidney stones through a magnetic “capture-and-carry” capability, functioning as an untethered soft grasper for artificial kidney stones. To evaluate the payload capacity, artificial stones with an average diameter of 2.54 mm and a mass of approximately 16.4 mg were utilized. As shown in Figure 6B (Movie S6), the droplet robot was remotely navigated to the target site, where it successfully engulfed the entire stone within only 1 s upon contact. During the retrieval phase, under the application of a dual-coil electromagnetic field (51 mT, 8.3 Hz), the magnetic gradient force generated was sufficient to overcome both the static friction of the stone against the substrate and the viscous drag of the fluid. Remarkably, the droplet maintained stable encapsulation and transported the solid payload at an average velocity of 13.45 mm/s. This successful execution highlights the platform’s potential for minimally invasive, non-contact stone extraction procedures in confined urological tracts.

Together, these results demonstrated that programmable electromagnetic actuation enables not only controlled motion but also functional task execution, highlighting the potential of ferrofluid droplets for biomedical applications such as targeted delivery and minimally invasive stone retrieval.

## 4. Discussion

Herein, we developed a multifunctional magnetic droplet robotic platform for urological applications. This programmable electromagnetic field could precisely modulate the performance of ferrofluid droplets, achieving targeted navigation, localized drug delivery, and stone retrieval within a confined, tortuous 3D urinary model, which mimics the complex physiological environment of the urinary tract. The deformable capabilities of the droplets allow them to pass through constricted spaces even smaller than their static diameter while maintaining structural integrity, offering a key advantage over rigid instruments by reducing the potential damage of healthy tissues. Specifically, this electromagnetic-actuated system can modulate the active locomotion of the droplets that overcome the normal voiding-induced washout effect and physiological barrier of the urothelium. As a consequence of these effects, the droplets can target lesion sites to deliver therapeutic drugs and execute stone retrieval. Additionally, the rapid drug release kinetics of the droplets enhances the contact time between the therapeutic drugs and the lesion sites, thereby improving overall therapeutic efficacy.

The consistency between finite element simulations and experimental observations confirms that the programmed magnetic field gradients can effectively drive droplet locomotion. The differences between the single- and dual-coil actuation modes highlight the flexibility and practicality of this system, where localized fields facilitate higher speeds, and distributed fields provide greater stability and control. This unique functional tendency renders the platform highly adaptable to various applications in the urinary system. The conventional electromagnetic fields are able to precisely control the linear movement, but it is difficult for the droplets to navigate along the predefined track [30,31,32,33]. Our programmable dual-coil array enables precise multi-directional navigation and extreme deformation (up to an aspect ratio of 38.5) within 1 mm constrictions, which represents a significant engineering advancement for navigating the highly constrained ureteropelvic junctions. In this study, the programmable electromagnetic system modulated the droplets’ not only precise movement toward the curved trajectory, but also stable navigation through narrow and highly constrained spaces. Furthermore, this extreme deformability in confined geometries parallels the transport behaviors recently explored in non-Newtonian and gel-like droplet systems [34,35,36]. However, while gel-like droplets typically rely on intrinsic viscoelasticity and shear-thinning properties to navigate narrow microchannels, our ferrofluid droplets achieve comparable morphological adaptation primarily through the active modulation of external magnetic gradient forces overcoming interfacial tension. This active structural reconfiguration provides an additional degree of freedom for navigating the highly complex and tortuous strictures of the human urinary tract.

Iron oxide nanoparticles are highly biocompatible in vivo and have been integrated into multiple Food and Drug Administration (FDA)-approved formulations, such as ferumoxytol for anemia treatment [37,38], kidney diseases [39], and various magnetic resonance imaging (MRI) contrast agents [40,41,42]. However, after these ferrofluid droplets deliver drugs, the persistent presence of high concentrations of iron oxide nanoparticles in vivo may induce cytotoxicity, such as damage to cellular metabolism and viability and the production of excessive reactive oxygen species [43,44]. In this study, the side effects of iron oxide nanoparticles were minimized through a post-intervention retrieval strategy. Upon completing located drug delivery, these ferrofluid droplets encapsulated stones and were rapidly retrieved by electromagnetic field control. This “deploy-treat-retrieve” closed-loop operational paradigm is a critical step forward in uro-bionics and the clinical translation of soft microrobotics, ensuring maximum therapeutic efficacy with minimal systemic footprints.

Despite promising urological applications, this study has several limitations that need further investigation. First, the current droplet generation process introduces size variability; future iterations will integrate microfluidic dispensing modules to ensure standardized droplet volumes. Second, the static fluid testing environment does not fully replicate the hydrodynamic shear forces of continuous urinary flow. To address this in future studies, dynamic flow-loop systems integrated with programmable peristaltic pumps will be employed to simulate both continuous basal urine production and intermittent high-velocity voiding events. To prevent the droplet robots from being washed away during such high-shear voiding simulations, a “magnetic anchoring” strategy can be implemented, wherein a strong, localized static magnetic gradient is temporarily applied to pin the ferrofluid firmly against the urothelial wall, effectively overcoming the fluidic drag force. Third, while the agarose hydrogel coating provides a suitable low-friction baseline, it lacks the complex biochemical composition of the native urothelium. In a protein-rich environment, proteins and mucins can rapidly adsorb onto the bare ferrofluid interface, lowering interfacial tension and potentially inducing droplet fragmentation under high shear forces. Future in vivo translation could utilize bio-inert fluorinated carrier liquids (e.g., perfluorocarbons) [45] or encapsulate the ferrofluid core within a muco-penetrating polymeric shell (e.g., PEG) [46] to shield the active oil interface from biofouling. Fourth, regarding the “capture-and-carry” capability, there is a strict geometric limitation for extracting rigid payloads. While the highly deformable unloaded droplet can navigate through a 1 mm constriction during the deployment phase, a rigid 2.54 mm stone cannot physically pass through such a stricture. Therefore, extracting oversized stones would necessitate standard clinical pre-fragmentation (e.g., laser lithotripsy) prior to droplet-mediated retrieval [47]. Most importantly, translating this platform to in vivo settings will require the integration of real-time imaging modalities, such as ultrasound or fluoroscopy, coupled with closed-loop visual feedback control algorithms to enhance actuation precision in non-transparent biological tissues [48]. Addressing these technical bottlenecks will pave the way for the clinical deployment of untethered magnetic soft robots in precision urology.

## Figures and Tables

**Table 1:** A comparison of the droplet and microrobotic locomotion velocities reported in the literature.

System	Actuation Method	Max Velocity (mm/s)	Environment	Reference
Liquid metal robot	Dynamic magnetic field	~200	Aqueous medium	Zhang et al. [27]
Liquid microrobot	Global magnetic actuation	~440	Liquid phase	Li et al. [28]
Ferrofluid droplet	Alternating magnetic field	~20	Microfluidic channel	Bijarchi et al. [31]
Ferrofluid droplet robot	Programmable electromagnetic coil array	~260	0.9% NaCl solution	This work

## Data Availability

The original contributions presented in this study are included in the article. Further inquiries can be directed to the corresponding author.

## Supplementary Materials

The following supporting information can be downloaded at https://www.mdpi.com/article/10.3390/mi17050569/s1, Table S1: The grid independence study and mesh convergence analysis for the double-coil electromagnetic actuation model; Table S2: The grid independence study and mesh convergence analysis for the single-coil electromagnetic actuation model; Movie S1: Comparison of ferrofluid droplet locomotion under single-coil and dual-coil electromagnetic actuation modes; Movie S2: The programmable multi-directional navigation of a ferrofluid droplet tracing a predefined “SBU” trajectory; Movie S3: The extreme morphological deformation of a ferrofluid droplet navigating through a variable-width constricted channel (from 7 mm to 1 mm); Movie S4: The precise spatiotemporal navigation of a ferrofluid droplet robot within an anatomically realistic 3D-printed urinary tract model; Movie S5: Targeted drug delivery via vibration-induced rapid release of encapsulated fluorescent dye; Movie S6: Magnetic “capture-and-carry” execution for the retrieval and transport of an artificial kidney stone.
