# Supplementary material for: Multifunctional Magnetic Droplet Robots for Urological Applications: From Drug Delivery to Stone Retrieval
Source: Micromachines (Basel). 2026 May 3;17(5):569. doi: 10.3390/mi17050569 (PMC13208772; doi:10.3390/mi17050569)
Supplement: Supplementary file 1 [file micromachines-17-00569-s001.zip › Supplementary File.pdf]

Supplementary File

Multifunctional Magnetic Droplet Robots for Urological Applications: From Drug Delivery to Stone Retrieval

Angelina Lin [1,2#], Joanna Tang[1,2#], Chunlian Zhong[1,2], Shanshan Yao[3], and Zhaoqing Cong[1,2*]

1 Department of Urology, Stony Brook University, Stony Brook, NY 11794, USA
2 Stony Brook Cancer Center, Renaissance School of Medicine, Stony Brook University, Stony Brook, NY 11794, USA
3 Department of Mechanical Engineering, Stony Brook University, Stony Brook, NY 11794, USA
# These authors have contributed equally to this work.
* Correspondence: e-mail: Zhaoqing.Cong@stonybrookmedicine.edu

Movie S1: Comparison of ferrofluid droplet locomotion under single-coil and dual-coil electromagnetic actuation modes.

Movie S2: Programmable multi-directional navigation of a ferrofluid droplet tracing a predefined "SBU" trajectory.

Movie S3: Extreme morphological deformation of a ferrofluid droplet navigating through a variable-width constricted channel (from 7 mm to 1 mm).

Movie S4: Precise spatiotemporal navigation of a ferrofluid droplet robot within an anatomically realistic 3D-printed urinary tract model.

Movie S5: Targeted drug delivery via vibration-induced rapid release of encapsulated fluorescent dye.

Movie S6: Magnetic "capture-and-carry" execution for the retrieval and transport of an artificial kidney stone.

Table S1. Grid independence study and mesh convergence analysis for the double-coil electromagnetic actuation model.

Mesh	Elements	DOF	Max B-field (T)	Final Droplet displacement (mm)	% change from previous
**Coarse**	3392	18304	0.252	0	
**Normal**	5052	26446	0.252	0	0%

**Fine**	7572	38772	0.252	0	0%
**Finer**	31940	155473	0.252	0	0%

Table S2. Grid independence study and mesh convergence analysis for the single-coil electromagnetic actuation model.

**Mesh**	**Elements**	**DOF**	**Max B-field (T)**	**Final Droplet displacement (mm)**	**% change from previous**
**Coarse**	3748	18572	0.252	11.8	
**Normal**	5456	26708	0.252	11.7	0.8%
**Fine**	8290	40011	0.252	11.8	0%
**Finer**	35738	166602	0.252	12.2	3.39%
